# Supplementary material for: Impact of COVID-19 Pandemic on (Health) Care Situation of People with Parkinson’s Disease in Germany (Care4PD)
Source: Brain Sci. 2021 Dec 31;12(1):62. doi: 10.3390/brainsci12010062 (PMC8773478; doi:10.3390/brainsci12010062)
Supplement: Supplementary file 1 [file brainsci-12-00062-s001.zip › brainsci-1517201-supplementary.pdf]

Supplementary Material

Table S1. Questionnaire

A1. COVID-specific questions – pLTC and np-LTC group

- Did you have a proven COVID-19 infection?

▢ Yes, with symptoms. ▢ Yes, without symptoms. ▢ No.

- Would you like to get the vaccination against COVID-19?

▢ Yes, I am already vaccinated. ▢ Yes, I wish to. ▢ Maybe. ▢ No.

- Did you recognize a worsening of your Parkinson's disease during the COVID-19 pandemic?

Not at all Very much

[0] [1] [2] [3] [4] [5] [6] [7] [8] [9] [10]

- Overall, how concerned are you about the COVID-19 pandemic?

Not at all Very much

[0] [1] [2] [3] [4] [5] [6] [7] [8] [9] [10]

- How strong is the impact of the COVID-19 pandemic on your everyday life?

No impact **Very strong**

[0] [1] [2] [3] [4] [5] [6] [7] [8] [9] [10]

- Have there been any phases during the COVID-19 pandemic in which you felt less supported in everyday life as compared to before?

Not at all Very frequently

[0] [1] [2] [3] [4] [5] [6] [7] [8] [9] [10]

- Which impairments did you perceive during the COVID-19 pandemic? Multiple answers possible.

▢ No impairment

▢ Less contact with or care by family/relatives/friends/third parties

▢ Less contact with or care by medical staff/doctors

▢ Less contact with or care by therapists (e.g., physiotherapy)

▢ Less contact with or care by nursing staff

▢ Not leaving the house/room

▢ Others, please specify: ………………………………………………………………

- Could you use telemedicine (telephone or internet-based visits) during the COVID-19 pandemic?

Not at all — Very frequently

[0] [1] [2] [3] [4] [5] [6] [7] [8] [9] [10]

- Would you like to use telemedicine (telephone or internet-based visits) on a regular base?

▢ Yes, but only additionally to personal doctor's visit.

▢ Yes, as an alternative to personal doctor's visit.

▢ No.

- Do you have the technical options to perform telemedicine?

▢ Yes, telephone & internet. ▢ Yes, telephone only. ▢ Yes, internet only. ▢ No.

A2. COVID-specific questions – p-LTC group only

- How well protected against COVID-19 do you feel by the sanitary measures of your professional care?

Not at all — Very well

[0] [1] [2] [3] [4] [5] [6] [7] [8] [9] [10]

- How well informed are you by your professional care about the current sanitary measures?

Not at all — Very well

[0] [1] [2] [3] [4] [5] [6] [7] [8] [9] [10]

- Which sanitary measures are used during your professional care sessions?

▢ Distance

▢ Hand hygiene (washing and disinfection of hands on a regular base)

▢ General hygiene measures (e.g., coughing / sneezing in the crook of the arm)

▢ Nursing staff wears a mask during care sessions

▢ Me, the patient, wears a mark during care sessions

▢ My family/relatives wear masks when come to visit me

▢ Limitation/interdiction of visitations

▢ Room ventilation

▢ Testing for COVID-19 in patients and/or nursing staff (e.g., PCR, quick tests)

▢ Don’ t know

▢ Others, please specify: ………………………………………………………………
